# Treatment of Ganglions

**Published:** 1871-02

**Authors:** 


Treatment of Ganglions.—Dr. Skey, of Bartholomew's Hospital, in a clinical lecture reported to the London *Lancet*, condemns the ordinary treatment of ganglionic swellings, which consists in giving a smart blow with a book or other body, and adds: "I advice you to adopt in great preference to this coarse and old-fashioned treatment the following, which rarely fails to obtain an early, if not immediate, cure. Its object is to evacuate the *entire* contents of the cyst, and to bring its opposite surfaces into perfect opposition with each other. It is a small operation; but on the delicacy of its operation its success materially depends. Bending the hand forwards, in order to tighten the skin over the cyst, pass vertically into the centre of the tumor a broad-shouldered lancet. By a lateral movement of the instrument the orifice will be dilated, and the contents will freely escape. Now, it is indispensable to the obliteration of the cyst that the whole of its contents should be evacuated—every drop and every fraction of a drop; to effect which the sac must be compressed and kneaded in every direction. Then apply a well-made, thick compress of lint, and strap it down tightly with good plaster, and, lastly, a roller may be applied. In 48 hours the wound has healed, and the ganglion is seen no more." —*Ibid.*

